# Spatial and Frequency Specific Artifact Reduction in Optically Pumped Magnetometer Recordings

**DOI:** 10.31083/j.jin2105145

**Published:** 2022-08-16

**Authors:** Jing Xiang, Han Tong, Yang Jiang, Maria E. Barnes-Davis

**Affiliations:** 1 MEG Center, Departments of Pediatrics and Neurology, Cincinnati Children’s Hospital Medical Center, University of Cincinnati, Cincinnati, OH 45229, USA; 2 Neuroscience Graduate Program, University of Cincinnati College of Medicine, Cincinnati, OH 45267, USA; 3 Department of Behavioral Science, University of Kentucky College of Medicine, Lexington, KY 40536, USA; 4 Perinatal Institute, Cincinnati Children’s Hospital Medical Center, Department of Pediatrics, University of Cincinnati College of Medicine, Cincinnati, OH 45229, USA

**Keywords:** magnetoencephalography, optically pumped magnetometer, artifact reduction, noise cancellation, signal space classification, time-frequency analysis

## Abstract

**Background:**

Magnetoencephalography (MEG) based on optically pumped magnetometers (OPMs) opens up new opportunities for brain research. However, OPM recordings are associated with artifacts. We describe a new artifact reduction method, frequency specific signal space classification (FSSSC), to improve the signal-to-noise ratio of OPM recordings.

**Methods:**

FSSSC was based on time-frequency analysis and signal space classification (SSC). SSC was accomplished by computing the orthogonality of the brain signal and artifact. A dipole phantom was used to determine if the method could remove artifacts and improve accuracy of source localization. Auditory evoked magnetic fields (AEFs) from human subjects were used to assess the usefulness of artifact reduction in the study of brain function because bilateral AEFs have proven a good benchmark for testing new methods. OPM data from empty room recordings were used to estimate magnetic artifacts. The effectiveness of FSSSC was assessed in waveforms, spectrograms, and covariance domains.

**Results:**

MEG recordings from phantom tests show that FSSSC can remove artifacts, normalize waveforms, and significantly improve source localization accuracy. MEG signals from human subjects show that FSSC can reveal auditory evoked magnetic responses overshadowed and distorted by artifacts. The present study demonstrates FSSSC is effective at removing artifacts in OPM recordings. This can facilitate the analyses of waveforms, spectrograms, and covariance. The accuracy of source localization of OPM recordings can be significantly improved by FSSSC.

**Conclusions:**

Brain responses distorted by artifacts can be restored. The results of the present study strongly support that artifact reduction is very important in order for OPMs to become a viable alternative to conventional MEG.

## 1. Introduction

Recent advances in optically pumped magnetometers (OPMs) have made it possible to provide a new type of magnetoencephalography (MEG), which is significantly different from conventional MEG [1–4]. In comparison to conventional MEG, which uses superconducting quantum interference devices (SQUID), that are cryogenic (−270 °C) as well as very costly and bulky, OPM MEG is non-cryogenic, cost effective and wearable. In fact, a helmet-style OPM MEG is flexible enough to place on a head of any size, which is important for clinical applications of MEG in pediatrics [5]. OPM MEG instrumentation can be contained inside of small cylindrical shields [6, 7] or may potentially be employed without shielding [2].

Neuromagnetic signals in MEG are typically very weak (e.g., 100–500 fT) and are easily overshadowed by artifacts (e.g., >3 pT) [8, 9]. Artifacts are unwanted interference in data that may mask desired signals. For MEG, artifacts are parts of the recorded signal arising from magnetic sources other than the neuromagnetic activity in the brain or the neuromagnetic signal of interest. Artifacts in conventional MEG systems typically include environmental, instrumental and biological artifacts [10, 11]. For OPM MEG, artifacts can also arise from the OPM themselves. Since the artifacts from the OPM sensors are generated by components within the OPMs (e.g., laser, heater, and vapor), these artifacts are called intrinsic artifacts [12, 13]. Although some artifacts appear within restricted frequency bands (e.g., powerline noise at 50 Hz or 60 Hz) and spatial subspaces (e.g., out of the head) [12, 13], many artifacts in OPM MEG may dynamically vary [13]. Consequently, it is essential to develop novel methods to remove artifacts in OPM MEG data.

Several methods for removing artifacts in traditional SQUID MEG systems have been developed (e.g., beamforming, and gradient noise cancellation) [8, 14, 15]. The majority of artifact reduction methods are based on a time-domain approach. A few frequency specific procedures for removal of artifacts have also been explored [15, 16]. To our knowledge, investigations of removal of artifacts in OPM MEG systems are scarce. A previous report [13] has experimentally evaluated artifacts arising from the laser intensity, laser frequency, laser polarization, cell temperature, and magnetic field coils used for the phase-sensitive detection of the magnetometer signal. Since the frequency ranges of the artifacts are unknown and may dynamically change, it is difficult to remove them in the time domain (e.g., we did not have the frequency edges to setup a band-stop filter to remove the artifacts). However, in Fourier space, we could easily remove the artifact by processing spectral data in each frequency bin. Of note, the interference-free frequency domain signals can be converted back to the time domain if necessary [15, 17]. To our knowledge, there has been no reporting on frequency-specific artifact reduction for OPM MEG recordings.

Artifacts in SQUID MEG data can also be reduced by using signal space separation (SSS) [18], dual signal subspace projection (DSSP) [11] and signal space projection (SSP) [9] methods. These methods are based on the fact that MEG data from multiple sensors can be classified according to their subspace. Specifically, MEG signals from the head and external artifacts from the sources outside of the head have different spatial patterns. These patterns can be modeled with spatial filters or beamformers. The spatial filters can be derived from source lead fields [19, 20]. Though signal space classification has shown promising results for artifact reduction in SQUID MEG [9, 11, 21], there has been only one report on spatial harmonic modeling with signal space projection in OPM MEG [22].

The objective of this study was to develop a new frequency specific signal space classification (FSSSC) method for removal of artifacts in MEG recordings. Different from previous reports on SQUID MEG [16, 23], this study specifically focuses on OPM MEG. To investigate artifacts in OPMs, we used commercially available OPMs [24, 25]. These OPMs are lightweight, wearable and work at room temperature. To use OPM MEG in pediatrics, we constructed a customized helmet in which OPMs are placed close to the scalp for detecting brain signals from a head of any size. We hypothesized that removal of artifacts in specific frequency bands and subspaces could enhance the identification of brain activation and improve the accuracy of source localization. To test the hypothesis, we assessed the performance of the artifact reduction methods with MEG data from a current phantom and from human subjects. It was anticipated that FSSSC in combination with the customized helmet might play an important role in applications of OPM MEG.

## 2. Materials and Methods

### 2.1 MEG Recordings

**Fig. 1.**
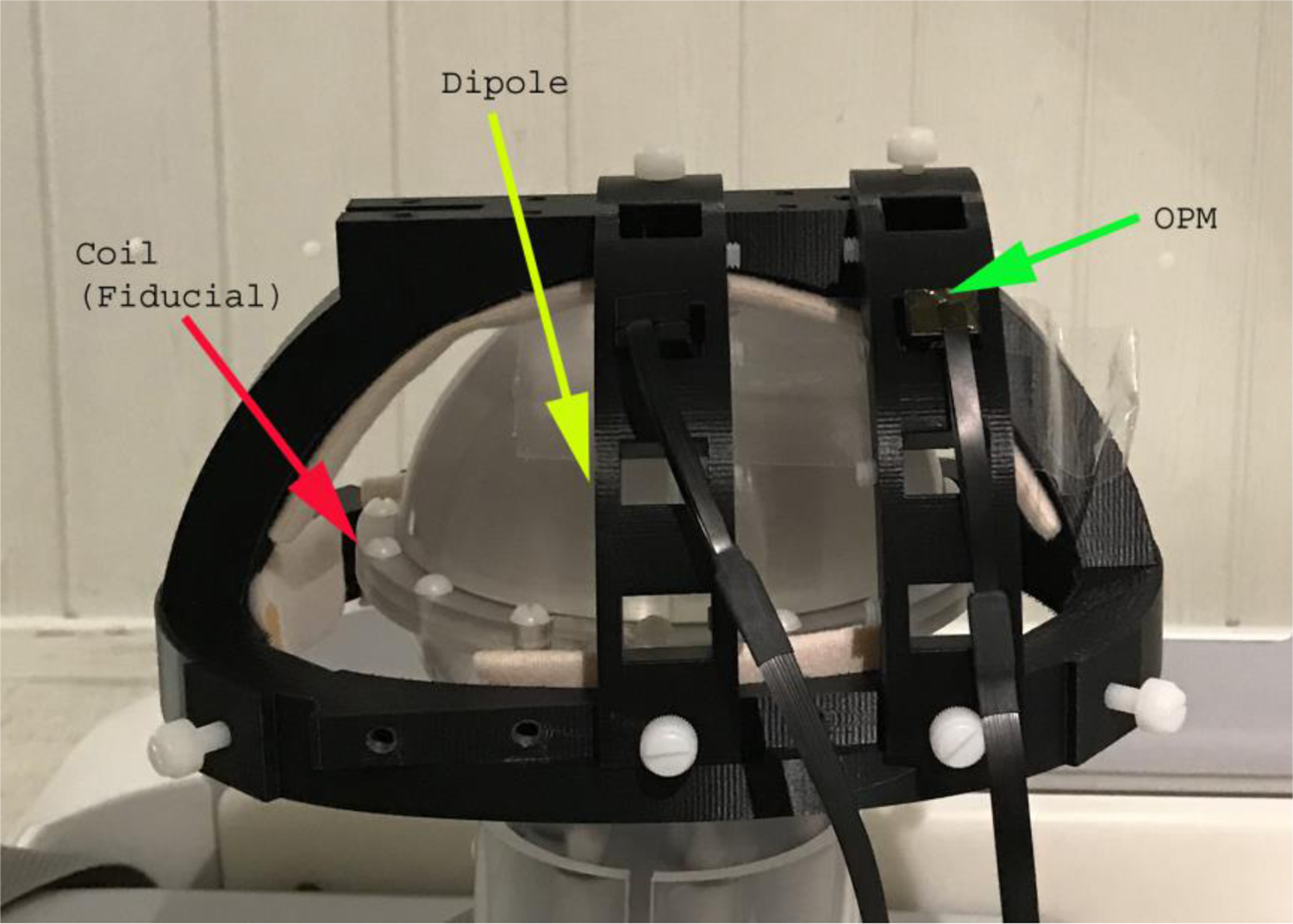
A photo showing the helmet, coils (fiducial points), source (dipole), and optically pumped magnetometer (OPM). The coil is used to localize the fiducial points relative to the position and orientation of sensors. The source generates predefined dipolar signals for developing and verifying methods (e.g., the location of the source is known). The OPMs are used to detect the signal from the dipole to assess the method for artifact reduction.

The MEG recordings were performed in a magnetically shielded room (MSR) (Vacuum-Schmelze, Hanau, Germany) [19, 20]. A portable MEG system was developed using 10 OPM sensors, a NI 9205 Data Acquisition Unit, and a notebook computer. The OPM sensors (Gen-2) were provided by QuSpin (QuSpin. Inc, Louisville, CO, USA) [4, 24]. The technical details have been described in previous reports [4, 24]. The field sensitivity of the sensor was <15 fT/√Hz (Z-direction) in the 3–100 Hz band (typically 7–10 fT/√Hz). The dynamic range was ±5 nT. A helmet was developed for holding the OPMs. The main frame of the helmet was 3D printed (Fig. 1).

The position of each OPM was adjustable for flexible detection of neuromagnetic signals from a desired brain region (e.g., the auditory cortex). Three coils were attached to the nasion and to the right and left pre-auricular points of each subject to locate the subject’s position head relative to the sensor position and orientation. Synchronized data acquisition from the OPMs was accomplished with a National Instruments (NI) card cDAQ-9171 (Austin, TX, USA). The OPMs (magnetometers) were linked to the NI card, which was then connected to a notebook computer (Surface 2.0, Microsoft Corporation, Redmond, WA, USA). A software package was developed to acquire data, perform dynamic artifact reduction and real-time signal processing. To identify system and environmental artifacts, two MEG datasets without phantom or subject were recorded just before MEG tests (empty room recordings).

### 2.2 Phantom

We used a current dipole phantom, which came with our CTF MEG system (CTF, Coquitlam, BC), to assess the performance of artifact reduction for the portable OPM MEG prototype [8]. A helmet was placed on the outside surface of the current dipole phantom for testing. The current dipole phantom was a spherical container filled with a conducting saline solution which contained a current source and sink. The globe had a 130 mm inner diameter. The current dipole itself was constructed of two gold spheres about 2.0 mm in diameter, separated by 9.0 mm centre to centre. The position of the current dipole could be adjusted within the globe. The location of the dipole was recorded relative to the center of the sphere (0, 0, 0 mm). The signal from the phantom was a sine waveform at 23 Hz. Two datasets were recorded without turning on the source (current dipole) before the phantom experiments to assess magnetic artifacts.

### 2.3 Human Participants

Six healthy subjects (age: 12–55 years, mean age: 30 ± 18 years; 3 males and 3 females) were recruited for this study. A written informed consent, approved by the Institutional Review Board at Cincinnati Children’s Hospital Medical Center (CCHMC), was obtained from each participant prior to testing. Similar to previous reports [26, 27], inclusion criteria for participation were: (1) healthy, without history of neurological disorder or brain injury; (2) normal hearing, vision, and hand movement. Exclusion criteria for participation included: (1) inability to sit comfortably for about 30 minutes; and (2) unidentifiable magnetic noise. Participants were instructed to listen to a 500 Hz square wave tone cue. The stimuli consisted of 200 trials of tones; 100 tones per ear, which were presented randomly through plastic tubes and earphones. Stimulus presentation and response recording were performed using BrainX software (Cincinnati Children’s Hospital Medical Center, Cincinnati, OH, USA), which was based on DirectX (Microsoft Corporation, Redmond, WA, USA) [26, 27]. Participants were instructed to sit comfortably and natural small movement was allowed. Two datasets without hearing tones were recorded before the experiments for analyzing magnetic artifacts.

### 2.4 Time Frequency Analysis

According to our pilot data from empty room recordings, each OPM had intrinsic artifacts itself (e.g., from the laser, vapor, and heater). Since the frequency ranges of the artifacts varied among OPMs and dynamically changed over recordings, it was difficult to remove them in the time domain (e.g., a band-stop filter requires the high and low frequency edges). However, in Fourier space, we could easily remove the artifact by processing spectral data in each frequency bin, the smallest frequency unit in spectrogram. To precisely remove artifacts in Fourier space, we transformed MEG data from the time domain to the frequency domain using the Fourier transform. Artifacts could be analyzed and identified by comparing experimental spectrograms (e.g., from phantom recordings) with empty room spectrograms. Once artifacts were identified and removed, we then transformed the frequency domain data back into the time domain for other data analyses [15, 19]. Before Fourier transformation, MEG data were high pass filtered with a corner frequency (CF) to remove slow variations at frequencies <CF for time-frequency analysis. The numbers of points (L) of the data sample were matched to the chosen CF, otherwise the MEG signal would be distorted and its amplitude diminished. A suitable matching condition was L ≥Sampling-Rate/CF or frequency resolution ≤CF [15, 19]. Instead of using spectral power (magnitude) for artifact reduction, the present study used the real and imaginary portion of data for artifact reduction so as to maintain the polarity information of the MEG data [19, 20].

### 2.5 Signal Space Classification and Artifact Reduction

**Fig. 2.**
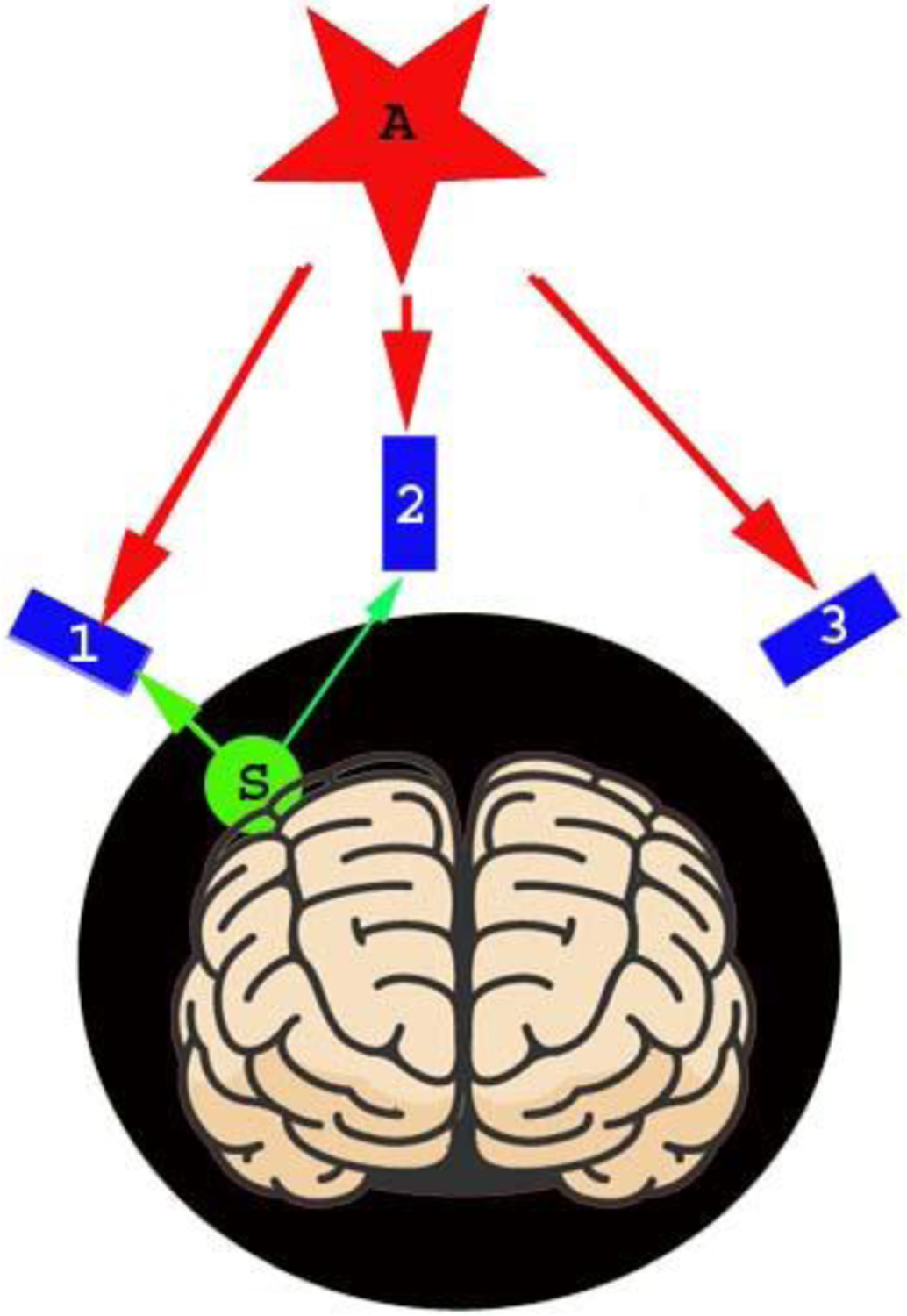
A diagram illustrating the spatial relationship between sensors, sources, and artifacts. All sensors are optically pumped magnetometers (OPMs) which can be placed and adjusted to cover a specific brain region (e.g., “1”, “2” and “3”). Sources are the signals of interest, which are from the brain in the head subspace (e.g., “S”). Artifacts are the noise and interferences from the environment and/or from the sensors (e.g., “A”), which are from the subspace out of the head.

Common mode artifacts (e.g., powerline noise, head movement) typically affect MEG data from all OPMs. Assuming MEG data include both brain signals and artifacts, we can partition the data into separate subspaces that originate from inside the head (brain signals) and outside the head (artifacts) [8, 11]. Fig. 2 shows the spatial relationship between brain signals and artifacts. Mathematically, we can determine basis sets used to distinguish between external signals and internal signals. In this study, MEG data from the OPM were therefore divided into outside MEG data (whose sources are out of the head) and inside MEG data (whose sources are within the head). The two types of data are supposed to belong to separate subspaces (Fig. 2).

Building on previous reports on SSS and DSSP [11, 18], the relationship between the measured signals B and artifacts can be described with the following equation.

(1) B(t)=Bi(t)+Bo(t)

where *t* represents time slices. *Bi(t)* represents inside signals from the brain. *Bo(t)* represents outside signals from artifacts. Brain signals can be estimated with lead field within the brain, which is the subspace of the head.


(2) Bi(t)=LQ(t)

(3) $$Q\left(t\right)=Bi\left(t\right)L^{+}$$

Where *L* is the lead field of sources (*Q*). Since the orthonormal signals of the source matrix *Q(t)* are assumed to be not related to source signals, these orthonormal signals are considered to be artifacts (*A*) [9]. The orthogonality of the brain signal (or the lead field) and artefact are mathematically calculated with cross-products (e.g., a lead field vector or a matrix of multi-lead field vectors). Consequently, MEG signals can be classified as two subspaces: brain signals and environmental artifacts. The brain signals can be obtained with the following equation.


(4) $$\hat{Bi\left(t\right)}=B\left(t\right)\left(I-AA^{T}\right)$$

Where $\hat{Bi\left(t\right)}$ represents estimated brain signals and *B(t)* represents measured signals. *A*^*T*^ represents the translated matrix of artifacts. I represents identity matrix. From beamforming point of view, $\hat{Bi\left(t\right)}$ represents the subspace of the sources (signal of interest); *AA*^*T*^ represents the subspace of the artifacts (interference). In the present study, the separation of sources and artifacts is called signal space classification (SSC).

Building on previous reports on artifact reduction in the time domain, we have derived our artifact reduction methods (Eqns. 3,4) [9, 11, 21]. In the present study, we moved one step further by removing artifacts in the frequency domain. Specifically, before artifact removal, we transferred MEG data from the time domain to the frequency domain. The removal procedure is based on a general input-output model with spectral data. Assuming a signal from a primary sensor contains both artifacts and brain signals; the relationship between the signal, brain activity and artifact can be described as the following equation.

(5) $$\hat{Bi\left(f\right)}=B\left(f\right)\left(I-A\left(f\right)A{\left(f\right)}^{T}\right)$$

where *B(f)* represents the raw signal in frequency domain, $\hat{Bi\left(f\right)}$ represents the brain signal in the frequency domain, and *A(f)* represents the matrix of artifacts in the frequency domain. I represents identity matrix. Our method includes a few novel options for artifact removal. Specifically, signals in certain frequency bands could be predefined for removal by analyzing spectrograms (e.g., artifacts identified in empty room recordings). For signals with variable frequency bands, the frequency bands were dynamically identified with SSC [15, 16]. A(f)A(f)^T^ represents the artifacts in the subspace of external signals, which are orthogonal to the subspace of interest signals (e.g., the brain) defined by lead field. Since the method enabled the removal of artifacts in a specific frequency band and subspace, this method was named frequency specific signal space classification (FSSSC). The method has been implemented in MEG Processor, which is publically available (https://sites.google.com/site/braincloudx/).

### 2.6 Performance Assessments

Shielding factor was used to quantify the level of suppression of artifacts. The algorithm for computing shielding factor for artifact reduction has been described in a previous report [28]. For waveform data, the shielding factor was estimated by calculating the ratio of the Frobenius norms of the measured and processed magnetic data. For frequency data, the shielding factor was the ratio of the magnitude (power) at a specific frequency band. Mathematically, shielding factor was computed with raw data and processed data. The raw data were OPM data, whose artifacts had not been removed with FSSSC. The processed data were OPM data, whose artifacts had been removed with FSSSC. The shielding factor can be obtained with the following equation.

(6) $$SF=\frac{\left|\left|\text{Bm}\right|\right|}{\left|\left|\text{Bp}\right|\right|}$$

where *SF* represents shielding factor; *||Bm||* represents the norm of the recorded raw data; *||Bp||* represents the norm of the processed data. The norm of data is the square root of the sum of each signal squared. From a MEG data analysis point of view, the shielding factor is equivalent to the signal-to-noise ratio.

### 2.7 Data Analysis and Source Localization

The waveforms of MEG data at sensor levels were visually inspected for distortion [19, 20]. MEG data were filtered with a high-pass filter of 3 Hz and low pass filter of 100 Hz before artifact identification. Frequency data were analysed in terms of power density. The covariance matrix was used to estimate the noise or signal correlation among sensors [19, 20]. The covariance matrix is an *N* × *N* matrix, where *N* is the number of sensors (channels) of the MEG recordings (*7* × *7*). The covariance matrix summarizes the spatial distribution in the sensor space of the noise or signal power (diagonal entries) and the spatial correlations between all MEG sensor recordings (off-diagonal entries).

Source localization was accomplished with beamforming [19, 20]. The grid spacing for the beamforming was 3 millimetres. We measured the location of the maximum activity and the distance from the maximum activity to the true sources (e.g., the current dipole in the phantom). The resulting artifacts and signals were used to estimate the source accuracy, which was quantified by directly calculating the distance between the estimated sources and the ground truth sources in a current dipole phantom.

### 2.8 Statistical Analysis

The normality of all values was checked using the Kolmogorov-Smirnov test. The comparisons of measurements (e.g., waveform amplitude) between raw data and processed data were performed using the *Student t*-test. The Bonferroni correction was applied for multiple comparisons. The significance level was set at *p* < 0.05. All statistical analyses were performed in R 3.6.1 and SPSS software package version 22.0.0.0 (IBM Corp, Armonk, NY, USA).

## 3. Results

### 3.1 Environmental and Intrinsic Artifacts

**Fig. 3.**
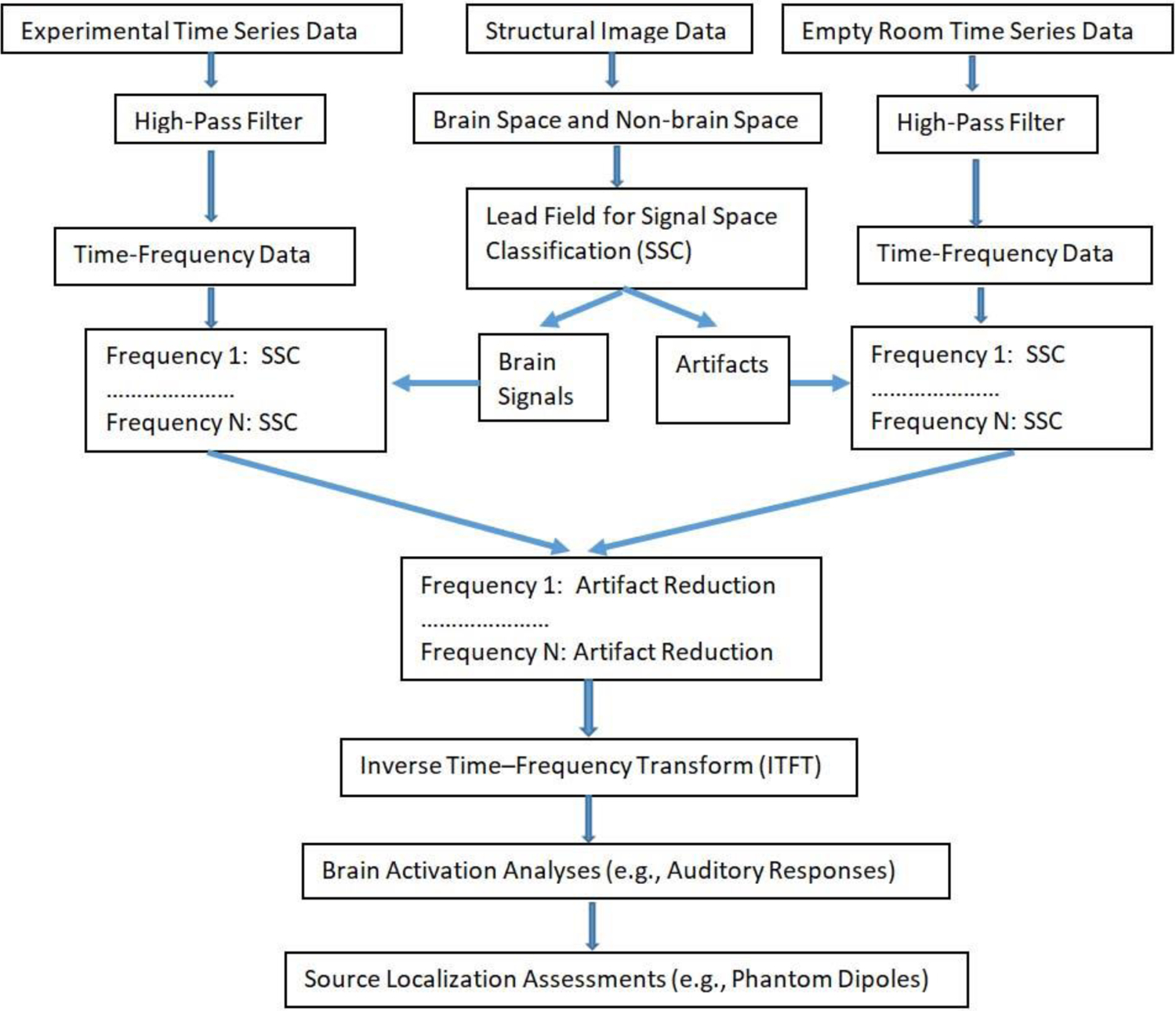
A block diagram of frequency specific signal space classification (FSSSC) for artifact reduction. The experimental time-series data include both brain signal and artifacts while the empty room time-series data mainly include artifacts. After performing time-frequency analysis and signal space classification, artifacts are removed and time-series data are computed for waveform analysis and source localization.

MEG datasets from empty room recordings without a subject showed environmental artifacts (background noise) and intrinsic artifacts associated with OPMs. Fig. 3 shows an example of waveforms from 7 OPM sensors. The amplitude of signals varied slightly among sensors and was dynamically changing with time. Since neuromagnetic signals from the brain are typically very weak (in a few hundreds of femtotesla), MEG signals with large amplitude (e.g., a few picotesla) are considered to be potential artifacts. The amplitude of one sensor (OPM2) was significantly higher than that of another sensor (OPM 3) (982 ± 171 fT vs. 704 ± 103 fT; *p* < 0.02) (see Fig. 3). After applying FSSSC, the processed data showed waveforms with low amplitude, which seemed to be stable and clean. Statistically, the difference between OPM2 and OPM3 was not significant after applying FSSSC (615 ± 42 fT vs. 590 ± 46 fT; *p* > 0.05). The shielding factor analyses revealed that frequency specific artifact reduction could significantly decrease the artifact in OPM MEG data (837 ± 158 fT vs. 638 ± 69 fT; *p* < 0.01).

**Fig. 4.**
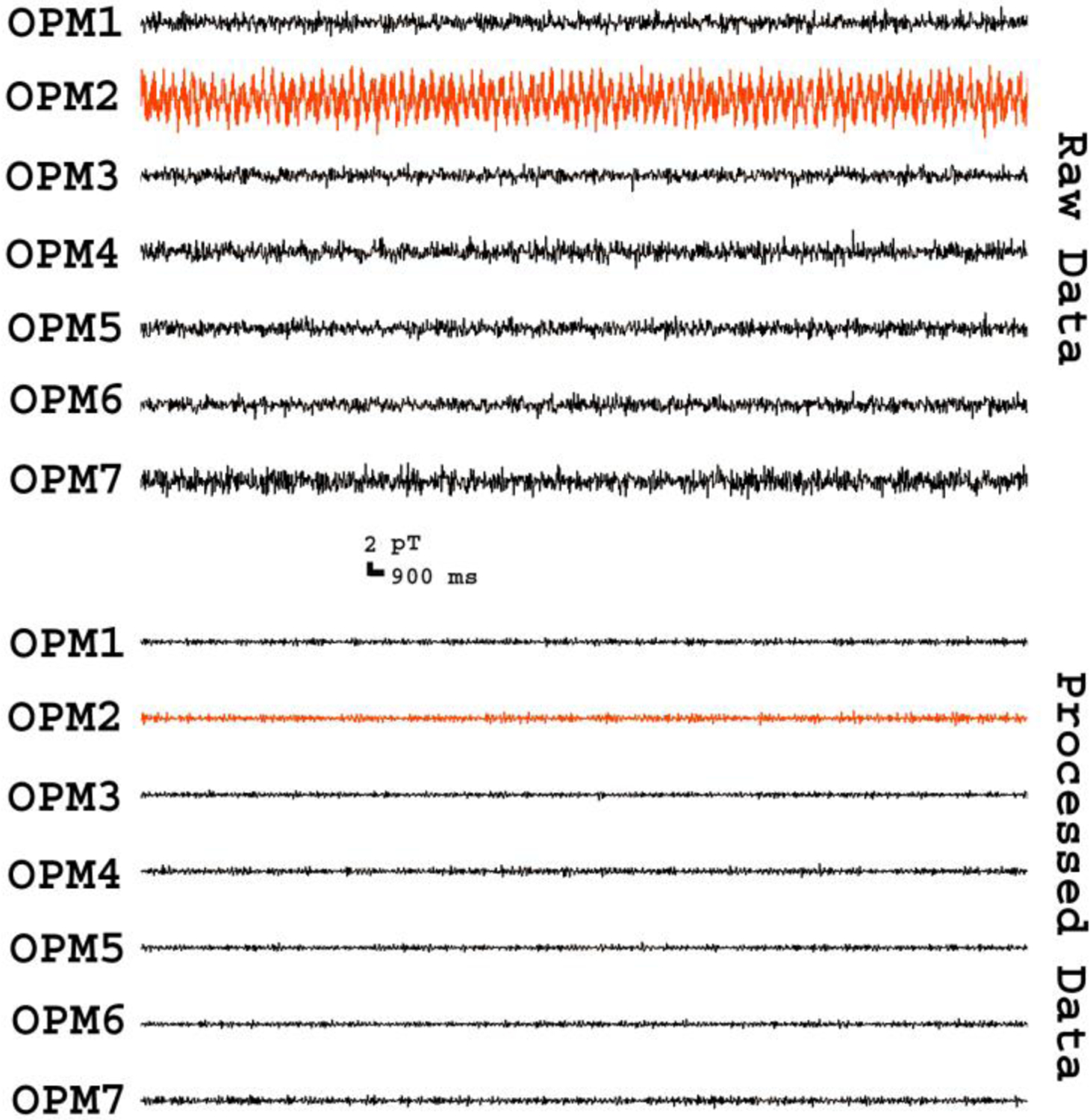
Segments of MEG waveforms from an empty room recording. The “Raw Data” show the waveforms of signals recorded from optically pumped magnetometers (OPMs) before artifact reduction. The “Processed Data” show the waveforms of signals after artifact reduction. In comparison to raw waveforms, processed waveforms have low amplitude and less variation among sensors (e.g., “OPM2” vs. “OPM3”). All waveforms are filtered with a bandpass filter of 3–100 Hz.

Time frequency analyses revealed artifacts in multi-frequency bands. The magnitude (spectral power) and frequency bands of some artifacts varied significantly among OPMs. The artifacts specifically associated with individual OPMs were considered to be intrinsic artifacts from the electronic constructing of the OPM (e.g., vapor, laser, heater, and wire connections). Fig. 4 shows one example of an intrinsic artifact around 8 Hz. After applying FSSSC, the processed data showed that the intrinsic artifact could be completely removed (Fig. 4). In comparison to spectrograms of raw MEG data, the spectrograms of processed MEG data showed less variation among sensors, and lower spectral power. The shielding factor analyses revealed that FSSSC could significantly decrease frequency-specific artifacts in OPMs. In the present study, the artifact reduction for signals around 7–9 Hz could attain a shielding factor of 160 (Fig. 3).

**Fig. 5.**
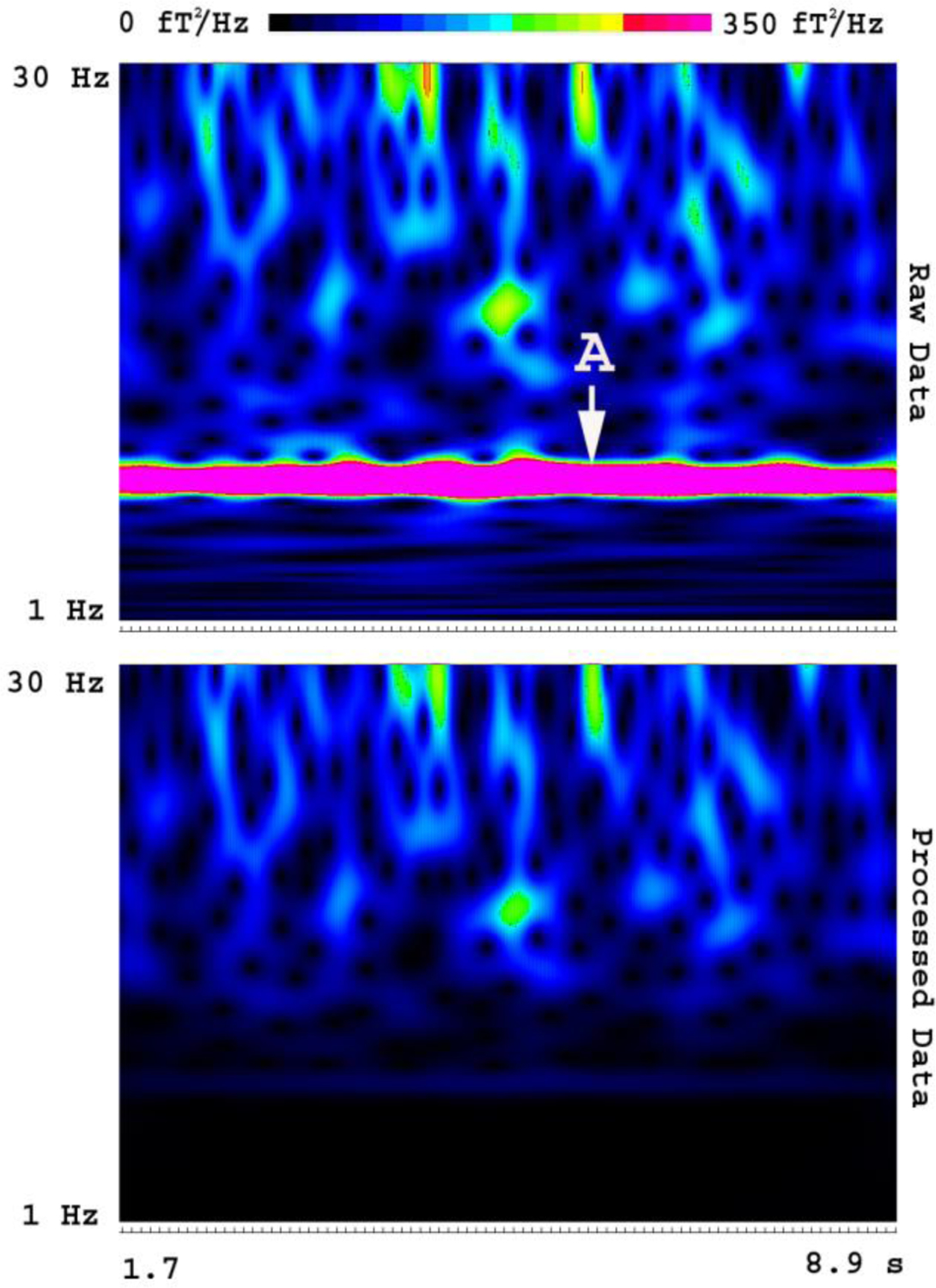
Spectrograms showing the removal of frequency specific artifacts. The “Raw Data” shows the time-frequency representation of MEG signals before artifact reduction. The “Processed Data” shows the time-frequency representation of MEG signals after artifact reduction. A frequency specific artifact (“A”) identified in raw data is completely removed in the processed data using FSSSC. Of note, the signals in other frequency bands are almost intact. The color bar indicates the color coding of spectral power, which is identical for the two spectrograms.

Fig. 5 shows an example of the artifacts in the frequency domain. We noted at least two types of artifacts. One type of artifacts appeared in a consistent frequency band in all OPMs (e.g., power line noise at 60 Hz and its harmonics). Another type of artifacts appeared in a few sensors (e.g., intrinsic artifacts). Artifacts identified on all OPMs appeared to be environmental (external) artifacts; the artifacts identified on a specific OPM seemed to be intrinsic (internal) artifacts. FSSSC developed in this study could remove both types of artifacts. Fig. 5 shows the removal of frequency specific artifacts in OPM data. In comparison to raw data, data processed with FSSSC showed a significant reduction in magnetic artifacts for all sensors. The shielding factor for reducing the artifacts reached 140 for artifact around 60 Hz.

**Fig. 6.**
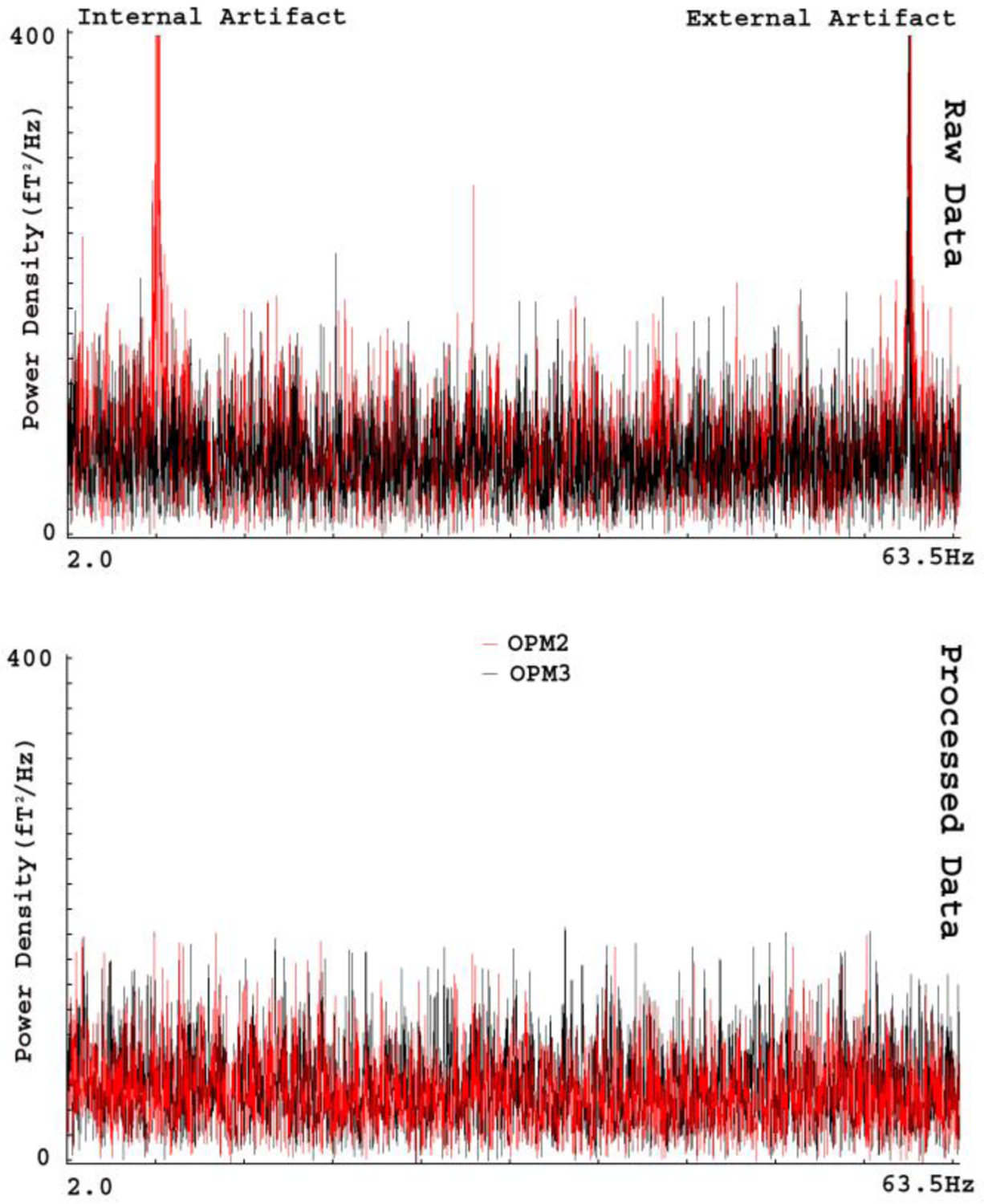
Power density revealing external and internal artifacts in OPM MEG data. The power density computed with raw data (“Raw Data”) shows two artifacts. The internal artifact (“Internal Artifact”) is predominantly identified in OPM2 but not OPM3. This observation implies the artifact is associated with the particular sensor. The external artifact (“External artifact”) is identified in all sensors (e.g., both “OPM2” and “OPM3”), which implies the artifact is from the environment and affects all sensors. The power density computed with processed data (“Processed Data”) show that frequency specific artifacts can be completely removed by FSSSC.

Covariance analyses revealed that some artifacts were correlated among OPMs. The correlated artifacts in OPM pairs were interpreted to be environmental artifacts. We noted that FSSSC removed sensor correlated artifacts and changed the covariance patterns. Fig. 6 shows an example of covariance matrices before and after the removal of correlated artifacts.

The results of waveforms, spectrograms, and covariance of empty room recordings revealed that artifacts in OPM data included external (environmental) and intrinsic artifacts. FSSSC removed both external (e.g., the artifact around 60 Hz) and intrinsic (e.g., 7–9 Hz) artifacts in certain frequency bands and subspaces (Figs. 2,3,4).

### 3.2 Phantom Data

**Fig. 7.**
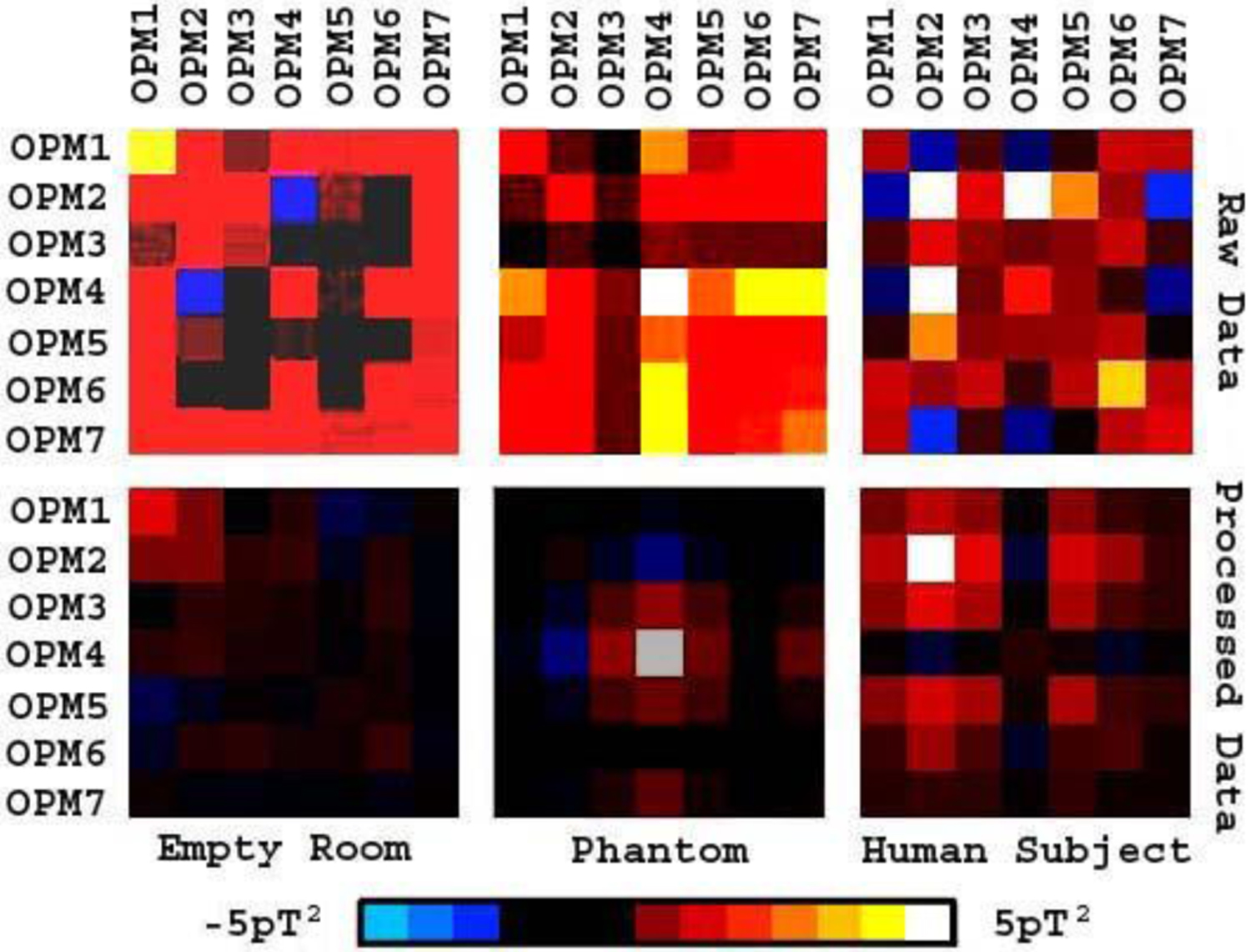
Covariance data showing the changes of the spatial distribution of signals for all sensor pairs. For data from the empty room recordings, the artifact reduction method significantly decreases the amplitude. For data from the phantom and a human subject, FSSSC significantly changes the patterns of covariance matrixes. The change of covariance pattern contributes to the improvement of source localization.

Source waveforms from the current dipole in the phantom were predefined as sine waves around 23 Hz. Fig. 7 shows the source waveforms and the recorded magnetic signals during experiments. The recorded magnetic waveforms were the source signals mixed with artifacts. There were significant differences between the source signals and the recorded signals in terms of the shape and phase of the waveforms. This was anticipated because artifacts from medical devices within a hospital setting could contaminate the experimental data (Fig. 7). The correlation between the source signals and the recorded signals in raw data was 0.51 ± 0.14. After processing with FSSSC, the signals of processed data were significantly improved and were similar to the source waveform (Fig. 7). The correlation between the source signals and processed signals was 0.76 ± 0.08. In comparison to raw data without artifact removal, processed data with artifact removal showed significantly stronger correlation with the source signals (0.76 vs. 0.61, *p* < 0.001). Since the source signals are the ground truth signals, the significant increase of correlation indicated that the FSSSC normalized and recovered the distorted signals in OPM MEG data.

**Fig. 8.**
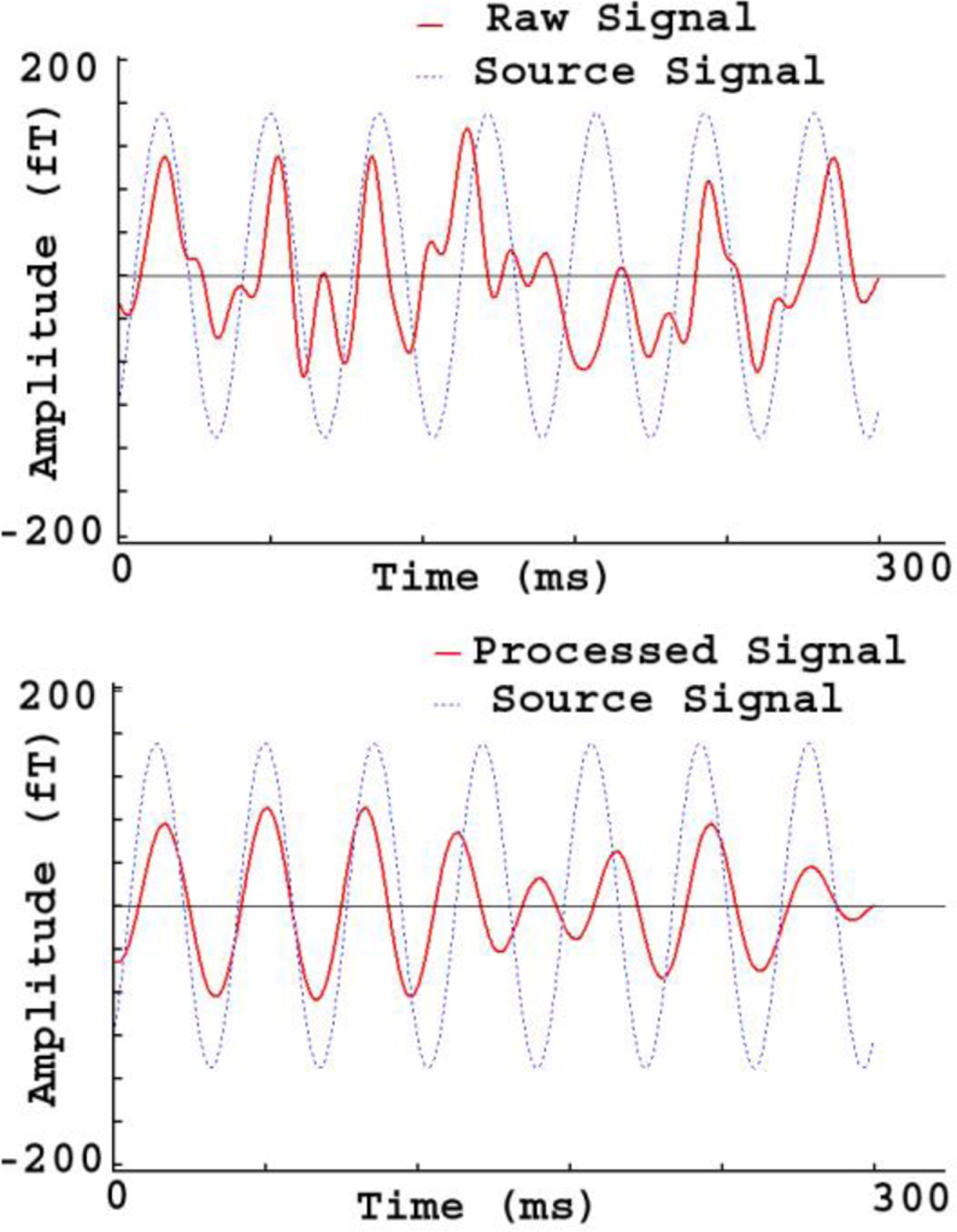
MEG Waveforms from a phantom source. The raw signal (“Raw Signal”) shows the source waveform distorted by artifacts. The processed signal (“Processed signal”) shows the source waveform with reduced artifacts. In comparison to the raw signal, the processed signal is much cleaner and is similar to the source signal in terms of wave shape and phase.

**Fig. 9.**
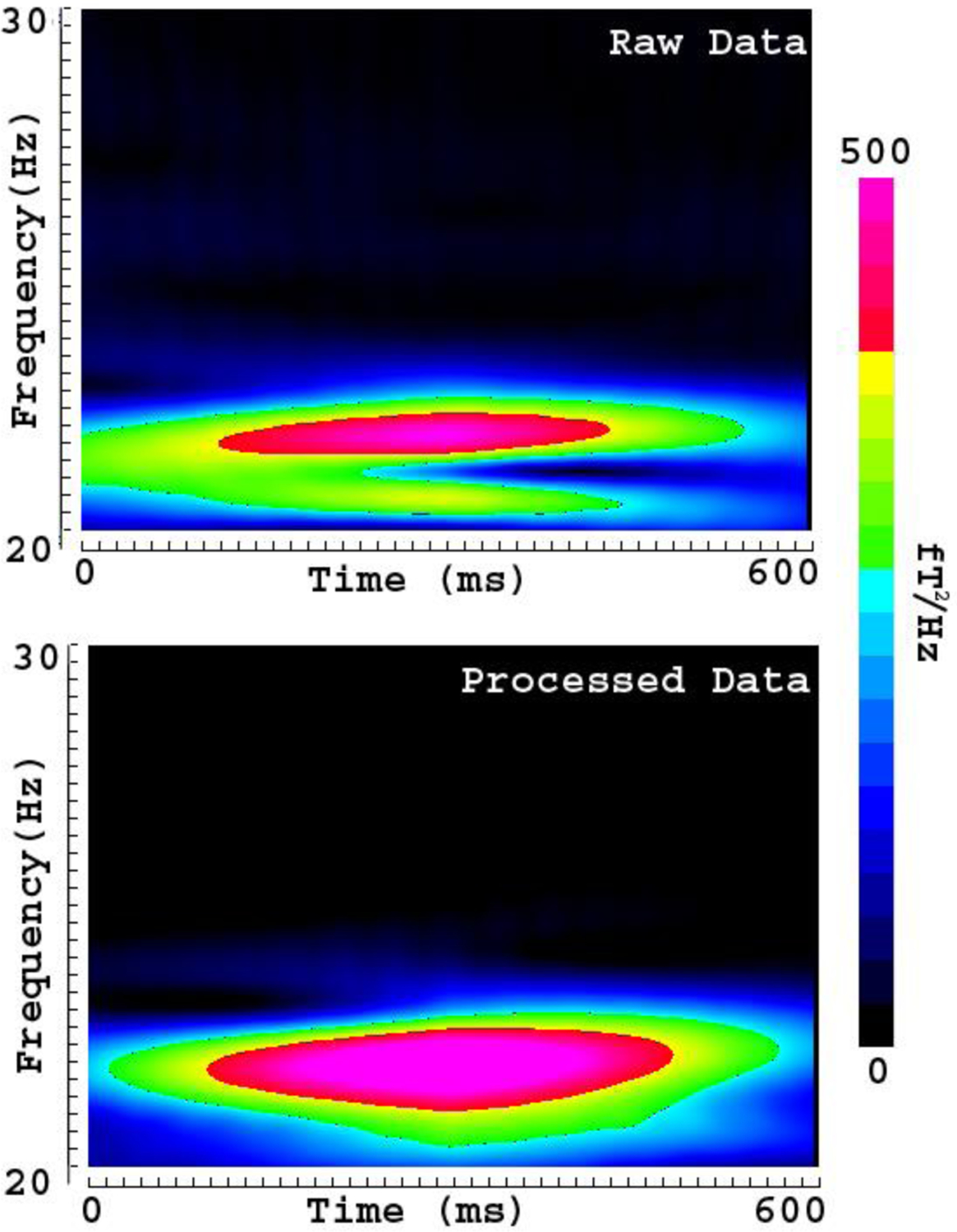
Spectrograms showing the time-frequency representation of phantom source and artifact. The spectrogram of raw data (upper panel, “Raw Data”) shows the time-frequency representation of the source signal distorted with artifacts. The spectrogram of processed data (bottom panel, “Processed Data”) shows the time-frequency representation of source signals after removing artifacts. A comparison of the raw and processed data indicates that removal of artifacts can recover and normalize source signals in the time-frequency domain. The color bar indicates the color coding of power density, which is identical for the two spectrograms.

Fig. 7 shows an example of covariance matrices of phantom data. Covariance matrix analyses revealed that FSSSC changed the spatial distribution. Fig. 8 shows the waveforms from a phantom source. Time frequency analyses revealed that the raw data had distorted frequency components, which were not from a single source signal. Fig. 9 shows an example of spectrograms computed for raw data and processed data. We noted that raw spectrograms included source signals and artifacts. After artifact removal (or reduction) processing, the processed spectrograms revealed a clear single frequency component which was at around 23 Hz.

The accuracy of source localization with raw OPM MEG data from the current dipole phantom was 4.97 ± 0.34 mm. The accuracy of source localization with processed OPM MEG data was 3.89 ± 0.072 mm. The source localized with processed OPM MEG data was significantly closer to the ground truth location, as compared to the source localized with raw OPM MEG data (*p* < 0.02). The results indicated that FSSSC significantly improved the accuracy of source localization.

### 3.3 Human Subject Data

**Fig. 10.**
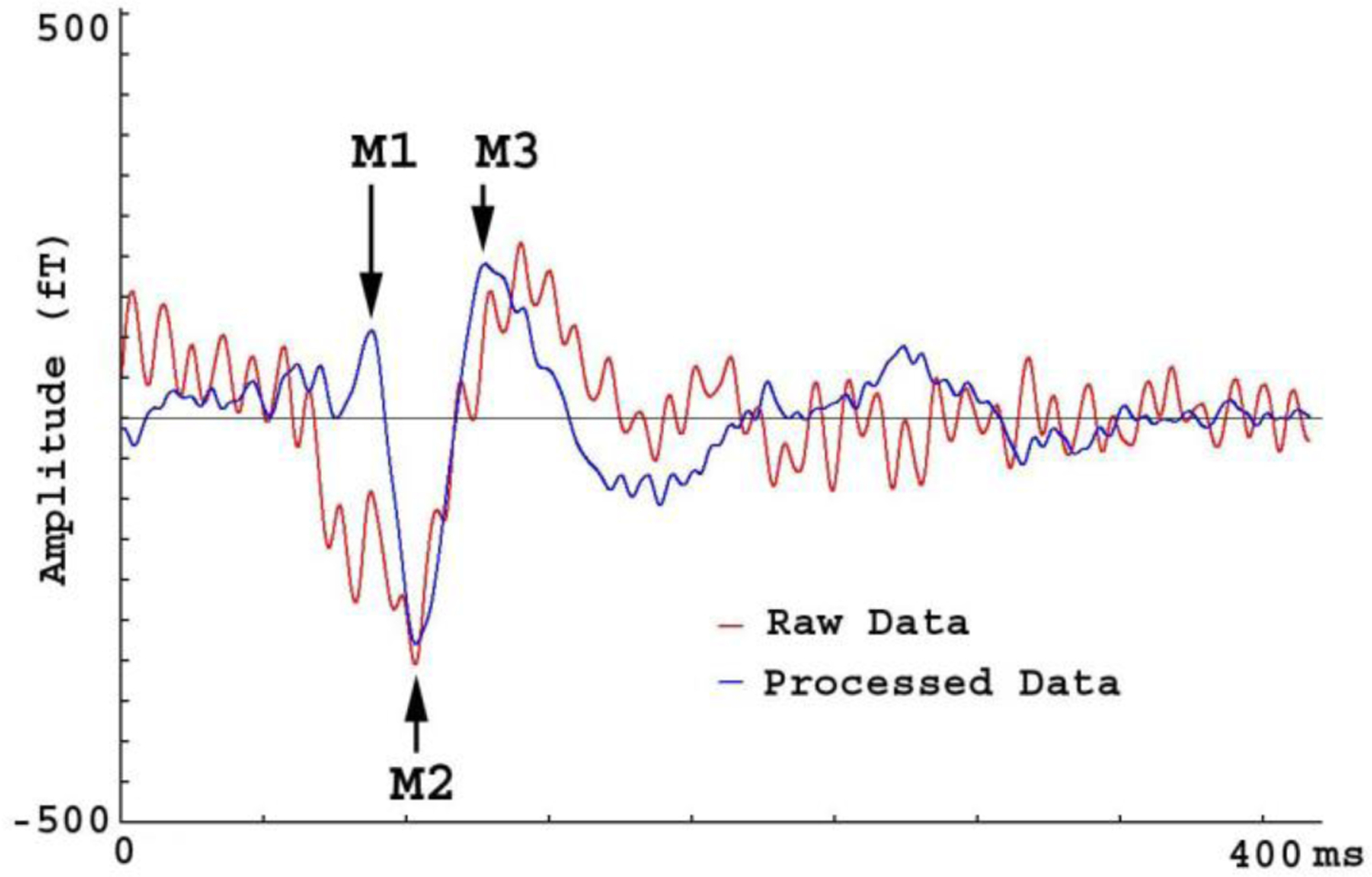
MEG Waveforms from a subject hearing a tone. The waveform of raw data (“Raw Data”) shows one deflection (M2) while M1 is completely obscured by artifacts. M3 is also lightly distorted by artifacts. The waveform of processed data shows three deflections (M1, M2 and M3) clearly.

**Fig. 11.**
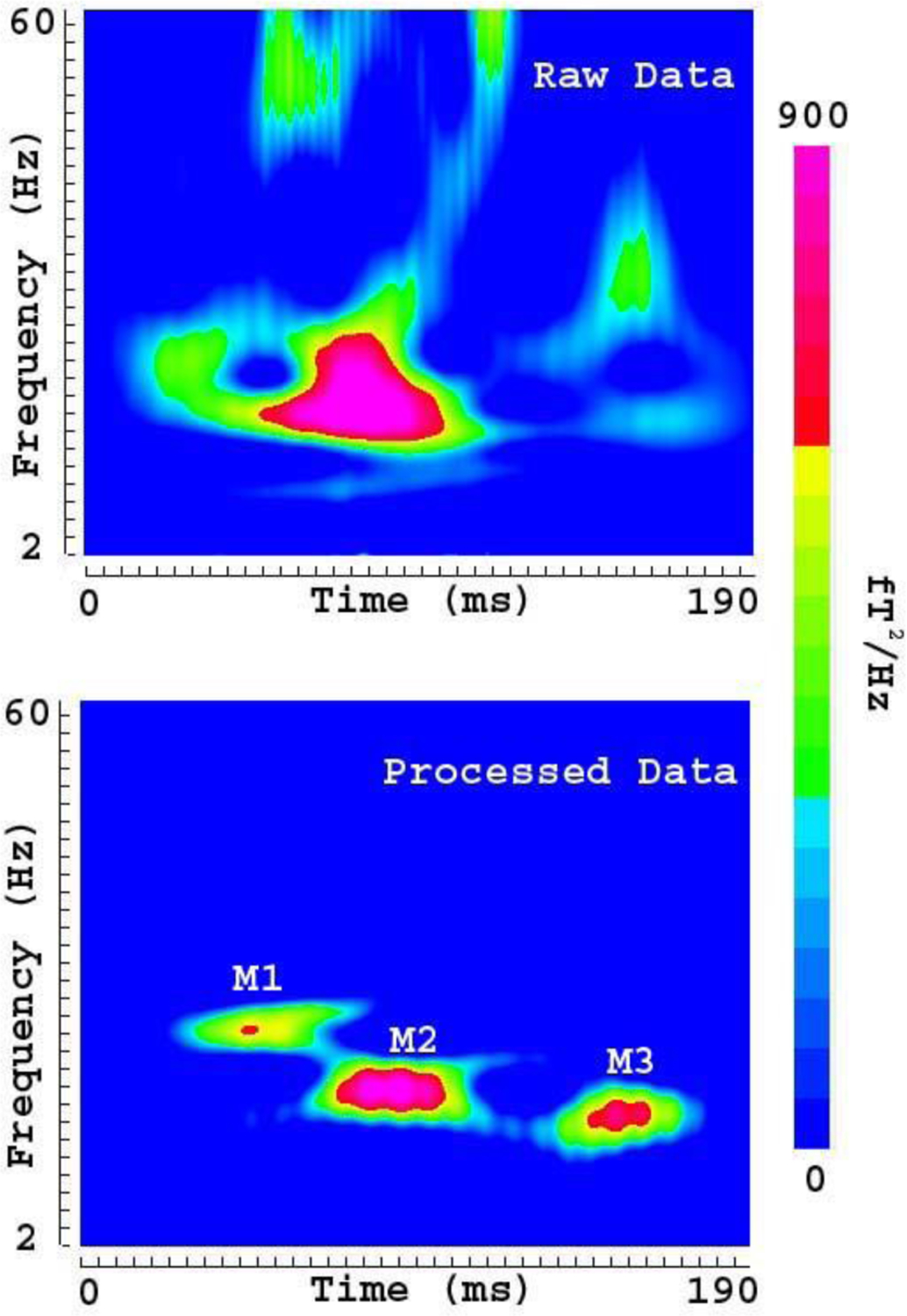
Spectrograms showing the time-frequency representation of auditory evoked magnetic fields (AEFs) and artifact. The spectrogram of raw data (upper panel, “Raw Data”) shows the time-frequency representation of the AEFs distorted with artifacts. The spectrogram of processed data (bottom panel, “Processed Data”) shows the time-frequency representation of AEFs after removing artifacts. A comparison of the raw and processed data indicates that removal of artifacts can reveal three components (M1, M2 and M3). The color bar indicates the color coding of power density, which is identical for the two spectrograms.

MEG data recorded from human participants revealed that OPMs recorded auditory evoked magnetic fields (AEFs). Fig. 10 shows the AEFs in raw MEG data and in processed MEG data. The strongest response in AEFs in SQUID MEG data was around 100 ms [27]. We also identified the strongest response in AEFs in OPM MEG data around 100 ms. This strongest response in AEFs was named M2 (>150 fT). In comparison to the raw MEG data, the processed MEG data also revealed a clear deflection around 70 ms (M1) and another deflection around 150 ms (M3). The M1 and M3 were well-studied in a previous report with a commercial SQUID MEG system [27]. We noted that the strongest AEF deflection was around 100 ms, which was clearly identifiable in raw MEG data. However, the weak M1 and M3 in AEFs (<150 fT) were only identified in processed MEG data. This observation indicated that the relatively weak M1 and M3 in AEFs in raw MEG data were overshadowed and distorted by artifacts. Time frequency analyses revealed one component in the raw data. However, time-frequency analyses revealed three components in the FSSC processed data (see Fig. 11). Figs. 10 and 11 demonstrate that FSSSC reveals these weak AEF components by removing or reducing artifacts.

Shielding factor analyses revealed that FSSSC removed artifacts by a factor of 8 for M2. The shielding factors for M1 and M2 were undetermined because the waveforms of M1 and M2 in the raw data were not clearly identifiable.

## 4. Discussion

The present study has established a good artifact reduction algorithm, FSSSC, for use with commercially available OPMs [4, 24]. Our preliminary results have demonstrated that OPM MEG can detect and localize relatively weak brain signals, as the conventional SQUID MEG does. Similar to the data recorded with SQUID MEG [15], data recorded with OPM MEG are associated with artifacts, which can be frequency- and space-specific. Some of the artifacts appear in a consistent frequency band while other artifacts appear in dynamically changing frequency bands [13]. The present study developed a FSSSC to selectively mitigate artifacts in frequency and space domains for OPM MEG recordings. The entire method consists of six steps: (1) Digital filter (e.g., remove low frequency drift); (2) Fourier transformation; (3) Spectral analysis (e.g., identify artifacts with empty room recordings); (4) Signal space separation; (5) Inverse Fourier transformation; and (6) Source localization.

Our time-frequency analyses have revealed that FSSSC can remove multi-frequency artifacts [12, 28]. Environmental noise appears in empty room recordings of the MEG data recorded without a human participant or a phantom. FSSSC can significantly mitigate the environmental artifacts in OPM MEG data. Since FSSSC is based on signal processing that can be accomplished with software, we postulate that software solutions can play a pivotal role in artifact reduction for OPM MEG in the future.

Artifacts in OPM MEG data also include intrinsic artifacts from components of the sensor itself (e.g., the laser, vapor, and heater) [7, 13]. The results of MEG data recorded from an empty room have demonstrated that artifacts are sensor-specific. For example, one of the OPMs in our study shows a much stronger artifact than other OPMs (see Fig. 2). An OPM is constructed with laser, vapor cell, heater, photodiode, photocurrent, electrical circuit, and other components that are used to heat the vapors. These components may generate magnetic artifacts that are related to the physical sensor [29, 30]. We consider magnetic artifacts from the OPM itself (e.g., laser, vapor, and heater) to be intrinsic artifacts. In the present study, the intrinsic artifact in each OPM was estimated with empty room recordings, which were free from artifacts from the human body or phantom devices. The artifacts associated with OPMs are common mode artifacts. These common mode artifacts may also be related to the system components, such as the electronics for controlling the OPMs and the helmet [4, 13]. FSSSC has demonstrated the capability to remove these common mode artifacts. Our findings are strongly supported by previous reports [15, 16].

One potential counter argument is that frequency-specific artifacts can be removed by using band-pass filters [8]. This argument is true for frequency-specific artifacts whose frequency bands are known and appear consistently in one or more frequency bands during the entire recordings. However, the frequency bands of artifacts in our OPM data are unknown. In addition, the frequency bands of the artifacts in the MEG data may change over time. Furthermore, according to our data, artifacts in OPM MEG data vary among OPMs and signal spaces. It would be troublesome for filtering variable artifacts among sensors and time with bandpass filters. Of note, FSSSC developed in the present study has been optimized for removing OPM artifacts by taking all these variables into consideration. Consequently, in comparison to band pass filters, FSSSC is much more powerful.

The removal of space-specific artifacts with FSSSC can also improve the accuracy of source localization. The results of phantom data analyses have demonstrated that the mitigating magnetic artifacts can normalize or recover the waveforms. The normalization of waveforms is visually identifiable by comparing MEG waveforms with the source waveforms (“gold standard” or “ground truth”). Consequently, the normalization of waveforms can change the spectrograms, power density, and covariance matrices. Since covariance matrices play a key role in beamforming [19], the source localization based on beamforming is also changed. By comparing the source location to the ground truth location of dipole in a phantom, we have found that FSSSC can significantly improve the accuracy of OPM MEG source localization. The results from the present study have demonstrated that it is important that the lead fields are also projected through FSSSC to correct for any spatial distortions it generates. The results of the phantom data indicate that the correction of spatial distortion is also important for deep sources (e.g., dipoles in the center of phantoms). It is reasonable to postulate that the localization errors from analysis of raw data are caused by the artifacts that lead to the distortion of MEG signals. It is important to point out that the distortion of signals is not only in waveforms but also in the spatial distributions of MEG signals (e.g., covariance matrix) [8, 11]. Consequently, the measurement of latency, amplitude, phase, and covariance of source waveforms are also distorted. Fortunately, FSSSC can normalize the waveforms, spatial distribution (covariance), and the latency and amplitude of the waveforms. Since MEG source localization (e.g., beamforming) is based on the covariance matrix of the signal patterns at the sensors [8, 31, 32], reducing artifacts can normalize waveform patterns that improve the accuracy of source localization. Thus, FSSSC can not only reduce the intrinsic and external artifacts in OPM MEG but also can improve the accuracy of source localization of OPM MEG, which is critical for pre-operative evaluation of epilepsy surgery in the future.

Artifact reduction is important for measurements of neuromagnetic activation recorded from human subjects [8, 11]. MEG data from human subjects have demonstrated that OPM MEG can capture AEFs. Since AEFs are originated from the left and right temporal lobes that require a good method to analyze the bilateral signals, AEFs are therefore a good benchmark and have been widely used for developing and testing new methods [33]. We have noted that the strongest component of AEFs is around 100 ms (M2). However, some weak responses (e.g., M1 and M2 components) can only be identified by using FSSSC. Since M1, M2 and M3 have been identified using conventional MEG [27, 34], to ensure that OPM MEG is viable alternative to conventional SQUID MEG, FSSSC or similar methods will be important for the development and wide-spread utilization of OPM MEG. Building on our previous studies with SQUID MEG [19, 20], the present study provides solid evidence that artifacts in OPM MEG can be significantly reduced with FSSSC. In fact, the waveforms processed with FSSSC are vastly better than the raw waveforms in the study of the auditory activation in the human brain. This is particularly true for weak neuromagnetic responses (Fig. 8, M1 and M3). Although signal space analysis has been used in OPM MEG [22], this is the first report showing the utility of FSSSC for suppressing the spatially and temporally-varying ambient artifacts in AEFs in OPM MEG. In contrast to previous reports [8, 32] focusing on the development of noise cancellation methods for conventional SQUID MEG systems, the present study focused on the development of new methods for OPM MEG systems. In comparison to conventional SQUID MEG systems, which are very expensive ($~3,000,000 USD), bulky, and cryogenic, OPM MEG can be cost effective (<$30,000 USD), be wearable, and work at room temperature. It is reasonable to postulate that the results from the present study strongly support that OPM MEG provides an alternative to the conventional SQUID MEG.

The capability of reducing external and internal artifacts in OPM MEG with FSSSC paves the way for clinical applications of OPM MEG. Since magnetic artifacts are dynamically changing in the hospital environment, we propose that our method can play an important role in clinical applications. In the present study, subjects were instructed to sit comfortably and natural small movement was allowed. This is different from conventional MEG tests, which requires participants to keep still. In pediatric studies with SQUID MEG [35–38], it is a challenge for children to keep still. From the clinical point of view, the OPM MEG employed in the present study is wearable, light-weight, and suitable to children with any head size. This is significantly better for pediatric studies than the conventional SQUID MEG systems, which are fixed, bulky, and not suitable for small head sizes. Our results support the argument that OPM MEG is a potentially important tool for pediatric research and clinical care, in that it might reduce or eliminate the need for general endotracheal anesthesia (GETA) often used to minimize movement artifact for pre-operative evaluation with SQUID-based MEG systems [39, 40]. According to our observation in the present study, OPMs combined with FSSSC can promote the applications of MEG in pediatrics and may avoid costly GETA for some patients in the future.

Though external magnetic artifacts can be reduced with hardware shielding, such as magnetically shielded room or Helmholtz coil lattices (active shield) [41, 42], these hardware shields limit the applications in a restricted environment that may not be practical in a clinical pediatric environment. Our approach is based on signal processing and software. The strength of our approach is that the operation can be applied to data without cost. Importantly, our solution can be turned off or on according to the user’s preference and purpose. If necessary, our method can be re-applied to MEG data without modifying the raw data or backup data. Thus, this software solution has unique strength compared to hardware shields or other solutions. Of course, a combination of hardware and software shield can be used together to significantly promote the applications of OPM in the future.

## 5. Conclusions

We have developed a new artifact reduction method, FSSSC, for use with OPM MEG. MEG results from the present study have demonstrated that artifacts in OPM MEG data can be reduced with FSSSC. FSSSC can normalize waveform, covariance matrixes, and spectrograms. Importantly, FSSSC can significantly improve the accuracy of source localization. AEFS identified by the conventional SQUID MEG can also be identified by OPM MEG with FSSSC. OPM MEG combined with FSSSC is much better than the conventional, immobile, and bulky SQUID MEG for pediatric applications because OPM MEG can be wearable and tailored for children with any head size. We propose this new artifact reduction method is not only compatible with but also complementary to other hardware and software solutions for improving OPM MEG signal.
